# Cardiogenic shock due to reverse takotsubo syndrome triggered by multiple sclerosis brainstem lesions: a case report and mini review

**DOI:** 10.3389/fcvm.2023.1175644

**Published:** 2023-05-03

**Authors:** Joas Brandner, Henri Lu, Olivier Muller, Elissavet Eskioglou, Jean-Daniel Chiche, Panagiotis Antiochos, Yaniv Chocron

**Affiliations:** ^1^ Service of Cardiology, Lausanne University Hospital, Lausanne, Switzerland; ^2^ Service of Neurology, Lausanne University Hospital, Lausanne, Switzerland; ^3^ Intensive Care Unit, Lausanne University Hospital, Lausanne, Switzerland

**Keywords:** takotsubo syndrome, multiple-sclerosis, takotsubo, reverse takotsubo, inverted takotsubo, cardiogenic shock, takotsubo (stress) cardiomyopathy, brainstem lesions

## Abstract

**Background:**

Takotsubo syndrome (TTS) is mainly characterized by chest pain, left ventricular dysfunction, ST-segment deviation on electrocardiogram (ECG) and elevated troponins in the absence of obstructive coronary artery disease. Diagnostic features include left ventricular systolic dysfunction shown on transthoracic echocardiography (TTE) with wall motion abnormalities, generally with the typical “apical ballooning” pattern. In very rare cases, it involves a reverse form which is characterized by basal and mid-ventricular severe hypokinesia or akinesia, and sparing of the apex. TTS is known to be triggered by emotional or physical stressors. Recently, multiple sclerosis (MS) has been described as a potential trigger of TTS, especially when lesions are located in the brainstem.

**Case summary:**

We herein report the case of a 26-year-old woman who developed cardiogenic shock due to reverse TTS in the setting of MS. After being admitted for suspected MS, the patient presented with rapidly deteriorating clinical condition, with acute pulmonary oedema and hemodynamic collapse, requiring mechanical ventilation and aminergic support. TTE found a severely reduced left ventricular ejection fraction (LVEF) of 20%, consistent with reverse TTS (basal and mid ventricular akinesia, apical hyperkinesia). Cardiac magnetic resonance imaging (MRI) performed 4 days later showed myocardial oedema in the mid and basal segments on T2-weighted imaging, with partial recovery of LVEF (46%), confirmed the diagnosis of TTS. In the meantime, the suspicion of MS was also confirmed, based on cerebral MRI and cerebral spinal fluid analyses, with a final diagnosis of reverse TTS induced by MS. High-dose intravenous corticotherapy was initiated. Subsequent evolution was marked by rapid clinical improvement, as well as normalization of LVEF and segmental wall-motion abnormalities.

**Conclusion:**

Our case is an example of the brain-heart relationship: it shows how neurologic inflammatory diseases can trigger a cardiogenic shock due to TTS, with potentially serious outcomes. It sheds light on the reverse form, which, although rare, has already been described in the setting of acute neurologic disorders. Only a handful of case reports have highlighted MS as a trigger of reverse TTS. Finally, through an updated systematic review, we highlight the unique features of patients with reversed TTS triggered by MS.

## Introduction

Takotsubo syndrome (TTS) is a transient structural heart condition associated with a surge of circulating catecholamines in the acute phase (1) and, in its typical form, characterized by akinesia of apical and mid-ventricular segments and hyperkinesia of basal segments, creating the typical pattern of “apical ballooning” found on cardiac imaging (cardiac ventriculography, transthoracic echocardiography (TTE) (1, 2). Initial presentation usually includes chest pain, ST-segment deviation on electrocardiogram (ECG) and elevated troponin levels (2), mimicking acute coronary syndrome, reason why the initial evaluation of patients with TTS typically includes a coronary angiogram to rule out significant coronary lesions (3). LVEF is generally decreased and clinical presentation can vary from mildly symptomatic systolic impairment to hemodynamic collapse, cardiogenic shock and, in the worst cases, cardiac arrest (4). No specific treatment has been developed to date and management mainly consists of symptomatic treatment and hemodynamic support if needed (3). Follow-up TTE exams usually show a spontaneous normalization of the wall-motion abnormalities and LVEF values (5). Typical triggers of TTS include emotional or physical stress (1). In some cases, an acute neurological condition, such as intra-cranial hemorrhage, stroke or seizures, has been described to cause TTS (1). Of note, in 28.5% of patients suffering from TTS no trigger could be identified (5). Recently, a few case reports have linked reverse TTS to multiple sclerosis (MS) (6–12). MS is the most prevalent chronic inflammatory demyelinating disease of the central nervous system (CNS) worldwide, and is characterized by partially or fully reversible events of neurologic disabilities, that in some cases can be rather asymptomatic. The course may be relapsing-remitting or progressive. Lesions in the CNS occur at different times and in different CNS locations (13).

In 2.2% of cases, TTS presents in a reverse fashion, in which wall-motion abnormalities are “inverted” (basal and mid ventricular akinesia, apical hyperkinesia), compared with the typical form (5). Around 80% of TTS patients are post-menopausal women, however, the population presenting with a reversed form of TTS is different: they are significantly younger with a mean age ranging from 57 to as low as 36 year old depending on the series (14, 15). Interestingly, the reversed form of TTS is significantly more frequent in patients with neurologic disorders (14). With this case report and literature review we want to highlight the link between MS and TTS and point up the atypical population suffering from TTS triggered by MS lesions.

## Case presentation

**Figure 1:**
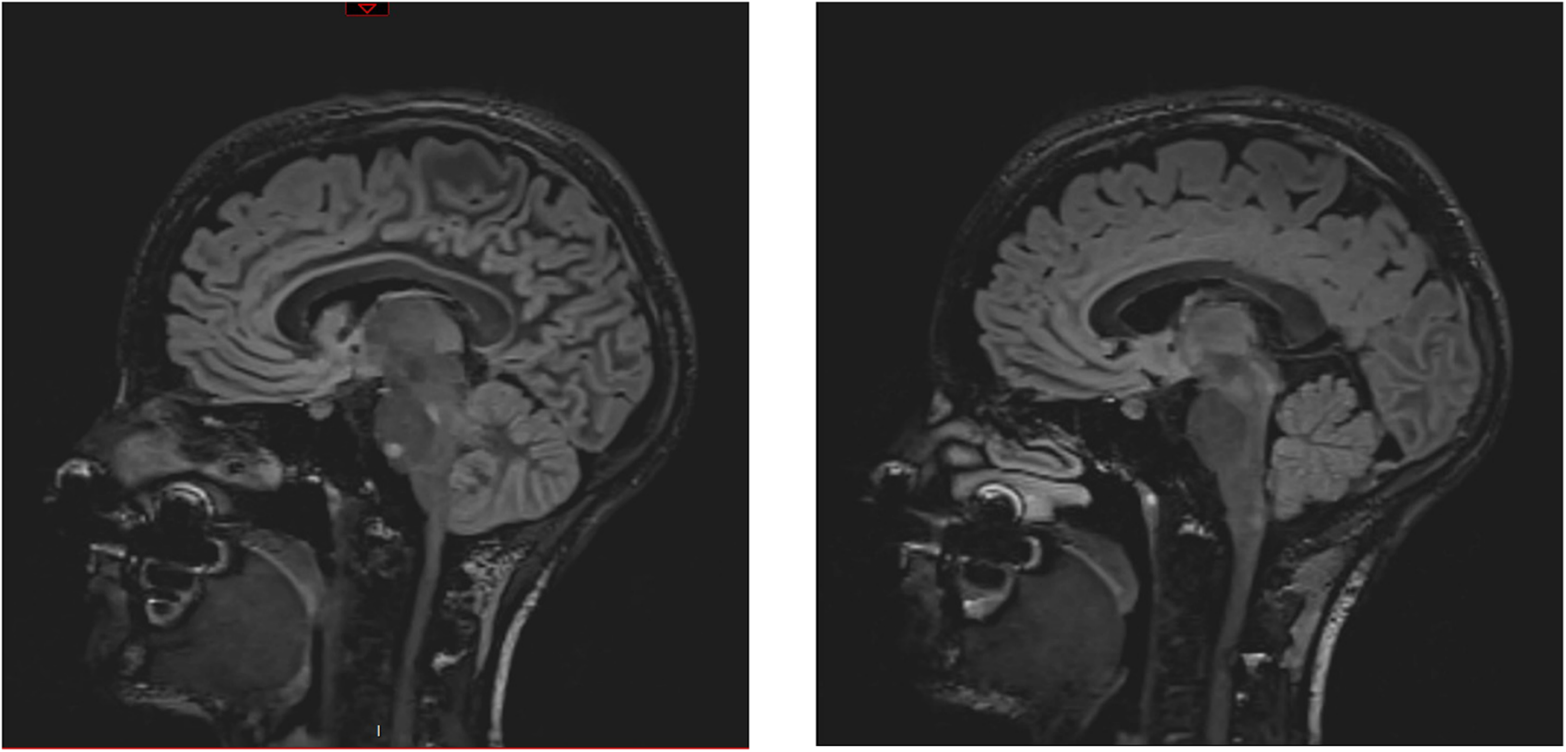
Cerebral magnetic resonance imaging (T2-flair) performed at day one showing three demyelinating in the brainstem.

**Figure 2:**
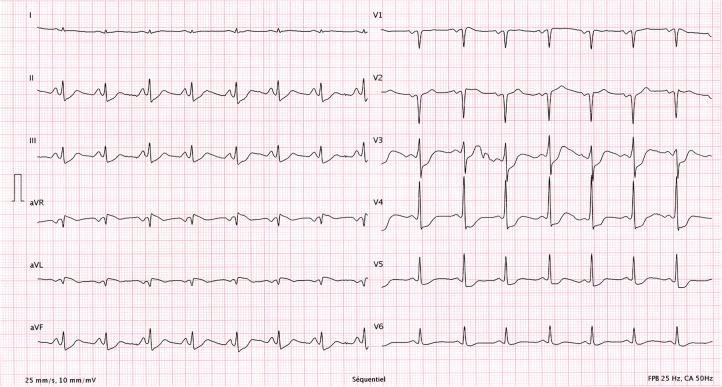
Electrocardiogram performed at day 1 revealing ST-segment depression in the inferior (II-III-aVF) and precordial (V3 through V6) leads.

An athletic 26-year-old woman was admitted to the emergency department of a Swiss secondary-level care hospital for new-onset numbness in the lower limbs. Initial neurological examination showed lower limb hypoesthesia up to the tenth dorsal level. Spinal magnetic resonance imaging (MRI) showed a T2 lesion at D12 level without contrast enhancement. Cerebral MRI revealed multiple demyelinating lesions, three of which were located in the brainstem (Figure 1: Cerebral magnetic resonance imaging (T2-flair) performed at day one showing three demyelinating in the brainstem). A lumbar puncture was performed and showed IgG oligoclonal bands with no other abnormality. Urinary pregnancy test was negative. At the time of her admission, apart from the neurological symptoms, she was asymptomatic and in a stable hemodynamic condition. During the course of the night, she was woken up by sudden unbearable holo-cranial pulsatile headache associated with hypertension, with systolic values up to 220 mmHg. She quickly developed chest pain, dyspnea and oxygen saturation dropped to 70% in ambient air. Initial arterial blood gases showed severe hypoxemia, with an oxygen partial pressure of 42 mmHg and lactic acidosis with a pH of 7.30 (N: 7.37–7.45) and an arterial lactate level of 5.3 mmol/L (N < 2.0 mmol/L). An ECG showed ST-segment depression in the inferior (II-III-aVF) and precordial (V3 through V6) leads (Figure 2: Electrocardiogram performed at day 1 revealing ST-segment depression in the inferior (II-III-aVF) and precordial (V3 through V6) leads). QTc time was measured at 400 ms. Serum high sensitivity (hs) T-troponins were elevated to 438 ng/L (N < 14 ng/L) and D-Dimers were measured at 20818 µg/L (N < 500 µg/L), without NT-proBNP elevation (value: 58 ng/L). Hematological testing was relevant for important leukocytosis of 23.2 G/l (N: 4–10 G/L) with 76% of segmented neutrophils as well as hemoconcentration with a hemoglobin level of 176 g/L (N: 120–157 g/L) and a hematocrit value of 0.52 L/L (N: 0.35–0.47 L/L). However, there was no evidence of active infection with a CRP level below the threshold of 5 mg/L and the absence of fever. The clinical condition worsened thereafter, with acute respiratory failure and hemodynamic instability, with sinus tachycardia (heart rate up to 150/min), requiring a transfer to the intensive care unit for urgent orotracheal intubation. During orotracheal intubation, blood pressure plummeted to a nadir of 59/44 mmHg, requiring aminergic support. Chest radiograph revealed acute pulmonary edema.

**Figure 3:**
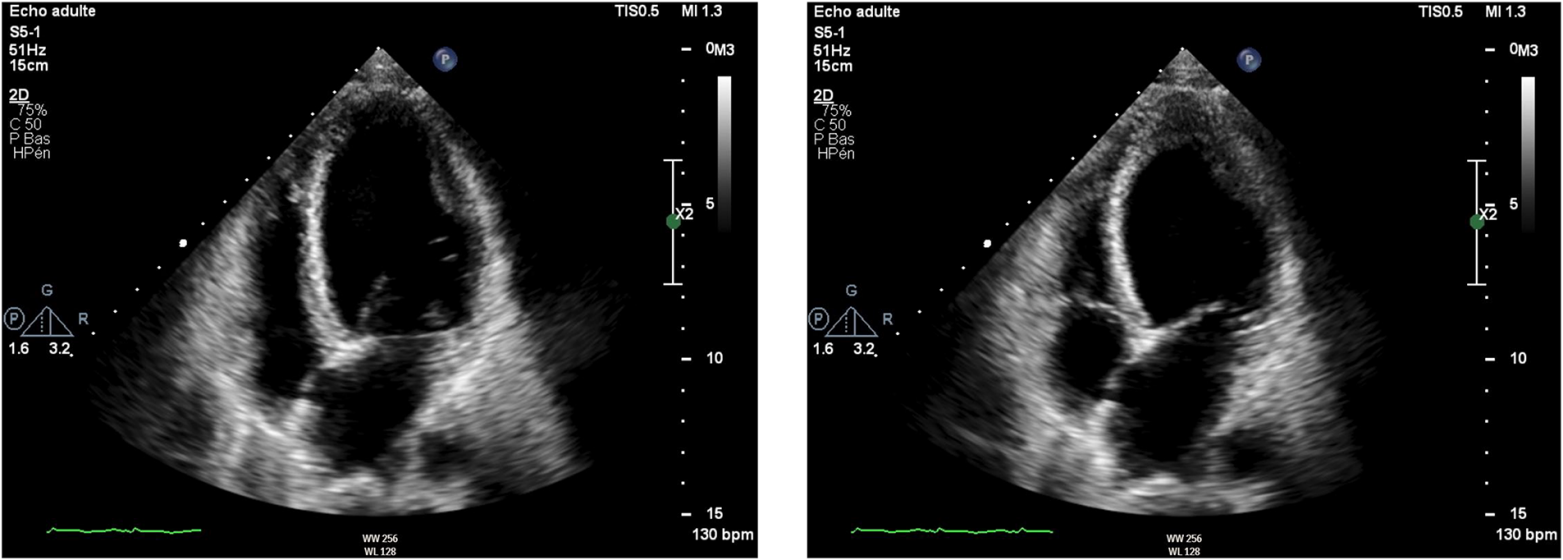
Transthoracic echocardiography performed at day one, showing a severely reduced left ventricular ejection fraction (24%) with basal and midventricular segments akinesia, and preserved apical contraction. There was no significant valvulopathy and right ventricular function was normal. **A**: Diastole, **B**: Systole.

**Figure 4:**
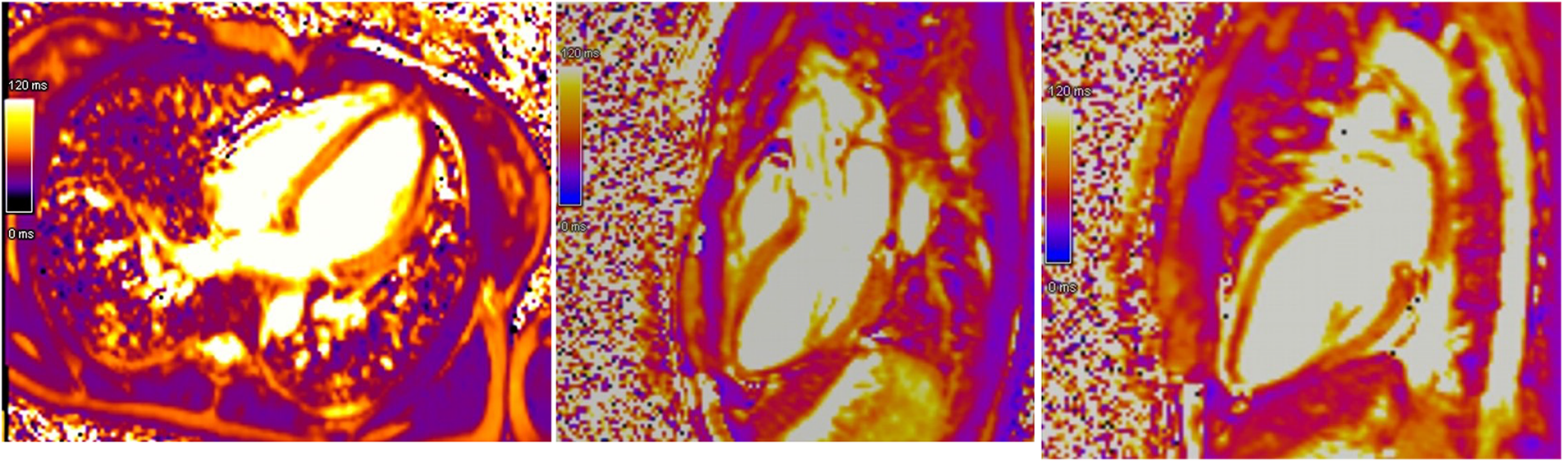
Cardiac MRI images from T2-mapping revealing myocardial oedema in four, three and two-chamber views.

Initial evaluation with TTE was suggestive of cardiogenic shock with an estimated LVEF of 15 to 20%. Because of the severity of the situation and the young age of the patient, a transfer to the academic tertiary care hospital was decided. Upon arrival, a total-body-CT was performed to rule out intracranial bleeding, pulmonary embolism, and an infectious or neoplastic process. TTE was repeated on day one and was relevant for severe LV dysfunction (LVEF of 24%), and findings suggestive of reverse TTS, with basal and mid-ventricular segments akinesia and a spared apical contractility (Figure 3: Transthoracic echocardiography performed at day one, showing a severely reduced left ventricular ejection fraction (24%) with basal and midventricular segments akinesia, and preserved apical contraction. There was no significant valvulopathy and right ventricular function was normal. Panel A: Diastole, Panel B: Systole). In the absence of localized wall motion abnormalities matching the territory of a coronary artery, and considering the young age of the patient, with no known personal or familial cardiovascular risk factors, coronary angiography was not performed as the probability of coronary artery disease was considered very low. Hs troponin T levels rose to a peak of 1,894 ng/L on day one, with a subsequent decrease. The clinical situation rapidly improved and the patient was extubated on day two. On day four, a cardiac MRI was performed, showing an improvement of the LVEF to 46%. Horizontal long-axis, color-coded images from T2-mapping revealed hyperintensity in the basal and mid-ventricular segments of the left ventricle that confirmed the presence of regional circumferential myocardial oedema (Figure 4: cardiac MRI images from T2-mapping revealing myocardial oedema in four, three and two-chamber views), as well as the absence of late gadolinium enhancement (supporting the idea that there was no irreversible myocardial injury) (16). Based on these observations, two diagnostic hypotheses were put forward: reverse TTS or an inflammatory cardiomyopathy. However, regarding the latter hypothesis, the Lake Louise criteria were not fulfilled (17), reason why reverse TTS was considered the most likely diagnosis, despite the patient having an InterTak Diagnostic Score of only 46 (25 points for female gender, 12 points for no ST-segment depression and 9 points for the acute neurological trigger), meaning a 9.8% probability of TTS (1).

On day two, cerebral and spinal MRI was repeated but showed no difference in the number of demyelinating lesions. On the basis of neurological clinical examination, MRI and lumbar puncture results, the diagnosis of MS was made by the neurology team, according to the 2017 revised McDonald criteria, as MRI demonstrated dissemination in space and the presence of cerebrospinal fluid-specific oligoclonal bands demonstrated dissemination in time (18). In the absence of any evidence for active infection, she was started on high-doses of intravenous corticoid therapy for 5 days for the treatment of MS, as per guidelines (19), with an excellent clinical response. The neurological symptoms progressively disappeared and at day nine the patient became completely asymptomatic.

**Figure 5:**
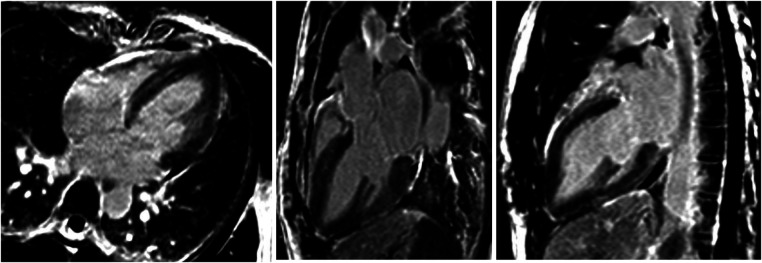
Absence of irreversible tissue injury (late gadolinium enhancement) in four, three and two-chamber views.

A subsequent TTE on day ten showed normalization of the LVEF to 63%, as well as the disappearance of the segmental wall motion abnormalities. The patient was discharged on day twelve without any treatment. Because of a rapid full cardiac function recovery, the hypothesis of an inflammatory cardiomyopathy as well as spontaneous coronary artery dissection was ruled out, confirmed by the follow-up cardiac MRI performed at three months, which showed the complete resolution of ventricular dysfunction. All these elements were in favor of a final diagnosis of reverse TTS triggered by brainstem lesions of MS (Figure 5: absence of irreversible tissue injury (late gadolinium enhancement) in four, three and two-chamber views) (16).

## Discussion

Acute neurologic conditions are known triggers of TTS. Inflammatory diseases of the CNS have already been described as potential causes of TTS. In fact, previous case reports have linked the specific and rare form of reverse TTS to MS. Furthermore, TTS is usually more frequent in post-menopausal women (5) and only 2.2% of TTS have been described to be reversed. Finally, no more than 5 to 10% of all TTS develop cardiogenic shock (5). In view of these elements, the case we describe is very unique, as the combination of all these factors (cardiogenic shock due to reverse TTS in a young woman, triggered by MS) has been rarely reported.

Clinical presentation of TTS often mimics acute coronary syndrome making the diagnosis challenging. According to InterTAK Diagnostic Score, developed by the European Society of Cardiology (1), seven features help in the diagnostic decision, including female gender, emotional or physical stress, neurologic disorder, or the absence of new ST-segment depression on ECG. Notably, around 15% of patients with TTS have concomitant coronary artery disease, making it unsuitable as an exclusion criterion of an acute coronary syndrome (5). This score can easily be used in the emergency setting and a score of 70 points (out of 100) yields a probability of TTS of 86%. Interestingly, in our case, the score was only 46 points, with a probability of 9.8% for TTS. Whether the fact that our patient presented with a very atypical form of TTS (i.e., reverse) partially explains this low score is unknown.

The pathophysiological mechanisms behind the genesis of TTS remain unexplained. To date, the main hypothesis is that of sympathetic stimulation related to an emotional or physical stress, inducing a catecholamine surge in the bloodstream, stunning myocardial contractility (1, 4). However, it remains unclear why this stunning selectively affects specific regions of the myocardium. It has been suggested that an apical higher density of ß-adrenergic receptors could explain this selectivity. Coronary microcirculatory dysfunction could also play a role (1). Furthermore, the overwhelming preponderance of women of postmenopausal age is highly suggestive of hormonal influence, specifically estrogen (1). A wide variety of triggers have been identified, first of which is emotional stress, ranging from depression to the news of a family member's death (1). Physical stressors have also been identified, most notably of neurologic origin, such as stroke, cerebral bleeding or cerebral concussion (1). Finally, some recently-published data also suggested a link between an oncologic process and TTS, as almost 10% of TTS patients seem to develop cancer in a 4-year follow-up (20). MS however has only been identified as the trigger of TTS in a few case reports across the scientific literature (5, 11, 12, 16), and even less so as the trigger of reverse TTS (7, 8, 10, 11, 21–27). To date, no specific treatment has been shown to improve clinical outcomes (3–5). Aminergic support should generally be avoided if possible as some studies suggested a higher mortality rate, with the possible exception of levosimendan (3). The standard of care is mostly based on close monitoring and treatment of potential complications. The latter can range from ventricular or atrial arrythmias to ischemic stroke due to ventricular thrombus caused by local akinesia and blood stasis. Up to 10% of total TTS patients develop cardiogenic shock and need hemodynamic support (5).

Regarding the specific condition of reverse TTS, it is defined by the same criteria as presented above, except for the segmental wall-motion abnormalities: akinesia affects the basal segments of the heart (instead of the apical segments, thus the words “reverse” or “inverted”), and may also involve the midventricular segments, but spares the apex (1, 4, 5). This rare form usually affects younger patients and has been described particularly in the setting of acute neurologic conditions, such as subarachnoid hemorrhage (1). Furthermore, reverse TTS seems to be associated with better clinical outcomes, compared with the traditional form of TTS (1, 5). Whether this better prognosis is due to the condition itself or to the fact that patients may be less comorbid (being younger), is not clear. A few case reports seemed to show a close association between MS and this particular form of TTS, highlighting the hypothesis of a special brain-heart connection.

Considering the relatively small number of case reports found in the literature, we conducted an updated systematic review to shed a brighter light on the association between reverse TTS and MS. The review was performed using PubMed/MEDLINE (Medical Literature Analysis and Retrieval System Online) and Embase (Excerpta Medica Database) online databases, from inception to 2022. Using Medical Subject Headings (MeSH), the following search strategy was used: “takotsubo” or “stress cardiomyopathy” AND “multiple sclerosis”. All case reports published in English were screened for the description of reverse TTS, either by TTE, cardiac MRI or ventriculography. Cases of TTS without a reverse form were excluded. 13 cases were eventually included (Table 1: Cases of reversed TTS in the setting of MS).

**Table 1:** Cases of reversed TTS in the setting of MS.

Patient	Clinical presentation, shock	TTE	Other examination	Treatment	Outcome	Reference
32-yo female	CV: atypical chest pain	Basal hypokinesia, 45% LVEF	MRI: ND	ND	Recovery	Reuss et al. (24)
	NL: ND		Angiography: NP			
29-yo male	CV: chest pain, hypertension	Basal and mid-ventricular hypokinesia	MRI: frontal lobe, vermis and pons MS lesions	ND	ND	Biesbroek et al. (8)
	NL: headache		Angiography: ND			
30-yo female	CV: chest pain	Basal and mid-ventricular hypokinesia	MRI: medulla oblongata MS lesions	3 days CS	ND	Kozu et al. (25)
	NL: headache		Angiography: ND			
43-yo female	CV: chest pain, dyspnea	Basal akinesia	MRI: ND	ND	Recovery	Peller et al. (10)
	NL: headache		Angiography: NP			
23-yo male	CV: dyspnea	Septal and anterior akinesia, 35% LVEF	MRI: medulla oblongata MS lesions	3 days CS	Recovery	Midaglia et al. (22)
	NL: headache		Angiography: ND			
16-yo female	CV: dyspnea, Cardiogenic shock	Basal hypokinesia, 35% LVEF, pericardial effusion, LV hypertrophy	MRI: medulla oblongata	Dobutamine, noradrenaline, ECMO, CS	Recovery	Androdias et al. (21)
	NL: hemiparesis, headache, diplopia, vomiting		Angiography: normal			
			CMR: normal			
25-yo female	CV: dyspnea, cardiogenic shock	Basal hypokinesia, 35% LVEF	MRI: medulla oblongata	Noradrenaline	Recovery	Androdias et al. (21)
	NL: paresthesia, headache		Angiography: normal	CS		
			Cardia MRI: normal			
27-yo female	CV: dyspnea, cardiogenic shock	Basal hypokinesia, 10% LVEF	MRI: medulla oblongata	Dobutamine, noradrenaline, ECMO	Recovery	Androdias et al. (21)
	NL: paresthesia, nystagmus		Angiography: normal	CS and plasma exchange		
			Myocardial biopsy: normal			
57-yo female	CV: chest pain, dyspnea	30% LVEF	MRI: medulla oblongata	ND	Recovery	Bayer et al. (26)
	NL: right facial numbness		Angiography: reduced function on ventriculography and basal hypokinesia with hyperdynamic apex			
30-yo female	CV: chest pain, palpitations, dyspnea, cardiogenic shock	Basal and mid-ventricular hypokinesia, 20% LVEF	MRI: medulla oblongata	Dobutamine, noninvasive mechanical ventilation, CS	Recovery	London et al. (7)
	NL: cerebellar ataxia		Chest x-ray: pulmonary oedema			
			Angiography: normal			
19-yo male	CV: dyspnea, hypertension	Basal hypokinesia, impaired LVEF	MRI: pontomedullary lesions	Nebivolol and candesartan, CS	Recovery	Rapp et al. (27)
	NL: left side paresthesia, nystagmus, unsteady gait		Cardiac MRI: no myocarditis			
			Angiography: normal			
42-yo female	CV: No symptoms documented	Inferior akinesia of the basal and mid-ventricular segments, 40% LVEF	MRI: medulla oblongata	ASA, ticagrelor, bisoprolol, CS	Recovery	Dell’Aquila et al. (11)
	NL: headache, upper limb hypoesthesia					
30-yo female	CV: atypical chest pain,	Basal akinesia and apical hypercontractility, 45%–50% LVEF	MRI: dorsal medulla	ND	Recovery	Cattaneao et al. (23)
	NL: bilateral proprioceptive ataxia		Angiography: normal			
26-yo female	CV: dyspnea, chest pain, hypertension, cardiogenic shock	Basal and midventricular akinesia, 15–20% LVEF	MRI: multiple brainstem lesions	Intubation, mechanical ventilation, CS	Recovery	Our case
	NL: lower limb bilateral numbness		LP: oligoclonal bands			
			CMR: basal and mid-ventricular hypokinesia and edema.			

Yo, year-old; TTS, Takotsubo syndrome; MS, Multiple sclerosis; TTE, Transthoracic echocardiography; CV, Cardiovascular; NL, neurologic; LVEF, Left ventricular ejection fraction; MRI, Magnetic resonance imaging; ND, Not documented; NP, Not performed; CS, Corticosteroid; LV, Left ventricle; CMR, Cardiac Magnetic Resonance Imaging; ECMO, Extracorporeal membrane oxygenation; ASA, Acetylsalicylic acid; LP, Lumbar puncture.

In the first case report from 2005, Reuss et al. reported three cases of TTS, among which the first known described case of reverse TTS in literature, in a 32-year-old woman presenting with MS (24). In 2016, Biesbroek et al. reported the second case, of a 30-year-old man developing reverse TTS in the setting of new-onset MS (8). This case, linking for the first time the two entities, led to a series of case reports showing an increasing interest in the association between reverse TTS and MS. Also in 2016, Kozu and colleagues reported the case of a 30-year-old woman with known MS presenting with chest pain, dyspnea and headache as well as well as hypertension (25). TTE revealed basal akinesia and MRI was relevant for an acute lesion in the medulla oblongata (25). In 2019, London et al. described yet another case of reverse TTS in a 30-year-old woman with relapsing MS in the medulla oblongata (7). In 2017, Androdias and colleagues published five cases of TTS (including 3 with the reverse form) associated with MS, in patients aged between 16 and 27, all of whom had brainstem lesions of the medulla oblongata (21). Finally, in 2020 Dell'Aquila et al. reported a last case of a 42-year-old woman with TTS and medulla oblongata lesions.

Further analysis of the cases showed that patients were in most cases young women, with a mean age of 30. We noticed a women/men ratio of 11/3 (78% were women). Five patients presented with cardiogenic shock, requiring inotropic agents, orotracheal intubation or even circulatory support with extracorporeal membrane oxygenation. Among these patients, 12 of them (85% of all patients) had lesions in the midbrain documented on MRI (9 of them, or 64% of all patients, in the medulla oblongata), thus upholding the hypothesis of a brain-heart connection mediated by the cardiac centers in this part of the brain. Notably, all documented cases seemed to have recovered from both their neurological symptoms and cardiac dysfunction, as documented by follow-up TTE.

All these cases suggest an association between reverse TTS and MS, and show how this population is different (at least 25 years younger) than the usual TTS population. One reason why reverse TTS is more commonly found in young woman may be related to the fact that they have been shown to present a larger number of adrenoreceptors at the base of the heart (thus being more prone to have reverse TTS), as compared to post-menopausal women, who have more adrenoreceptors at the apex (15). From a pathophysiological perspective, the actual understanding is that MS could be responsible for lesions in the cardiovascular centers of the brainstem (i.e., nervous centers located in the medulla oblongata, particularly in the solitary nucleus, that control heart rate, myocardial contractility through nervous and endocrine systems), with subsequent severe dysregulation of sympathetic activity leading to a catecholaminergic surge and secondary TTS (11). Considering the particular population which was affected by MS induced reversed TTS, further research should be considered to understand the brain-heart link and the extension to which MS affects the heart. Finally, in young patients suffering from reverse TTS without identification of a clear trigger, we suggest to actively look for potential existing or passed neurologic symptoms and in this case, consider MRI of the CNS and/or lumbar puncture.

## Conclusion

Our case is another example to the growing list of cases of reverse TTS associated with MS reported so far, all of which suggest a link between the two conditions. It shows how severe reverse TTS can be, with initial cardiogenic shock triggered by MS. Hopefully, it will help raise awareness on cardiac complications associated with this neurologic setting and increase interest in further research on the heart-brain axis. Furthermore, future research needs to be directed on the extent at which MS medication could prevent the recurrence of TTS. Finally, knowing that no etiology could be found in 28.5% of TTS patients, we encourage clinicians to actively search for MS in patients with reverse TTS without clear trigger, especially if they are young.

## Data Availability

The original contributions presented in the study are included in the article, further inquiries can be directed to the corresponding author.
